# Correlation between liver volume drainage and clinical success after endoscopic biliary drainage of hilar malignant obstruction

**DOI:** 10.1016/j.clinsp.2024.100540

**Published:** 2024-12-02

**Authors:** Jennifer Nakamura Ruas, Ernesto Quaresma Mendonça, Luciano Lenz, Gustavo Andrade de Paulo, Ricardo Uemura Sato, José Jukemura, Ulysses Ribeiro Junior, Fauze Maluf-Filho, Bruno Costa Martins

**Affiliations:** a Division of Endoscopy, Instituto do Câncer do Estado de São Paulo, São Paulo, SP, Brazil; b Department of Gastroenterology of Universidade de São Paulo, São Paulo, SP, Brazil; c Division of Gastrointestinal Surgery, Instituto do Câncer do Estado de São Paulo, São Paulo, SP, Brazil

**Keywords:** Cholestasis, ERCP, Common Bile Duct Neoplasms

## Abstract

**Background and aim:**

Malignant hilar obstruction usually presents in advanced-stage disease with a poor prognosis. Effective biliary drainage is essential for the beginning of palliative chemotherapy. There is a debate on the amount of liver parenchyma that should be drained to achieve clinical success. This study aimed to correlate the volume of liver drained with clinical success rate.

**Methods:**

The authors conducted a retrospective study including patients with malignant hilar biliary obstruction who underwent retrograde endoscopic cholangiography for biliary drainage from January 2014 to December 2018. The main outcome was a correlation of clinical success rate with hepatic volume drained. Secondary outcomes were correlation of clinical success rate with the quantity of liver sectors drained and unilateral versus bilateral drainage.

**Results:**

82 patients met inclusion criteria (58.5 % female), with a mean age of 60±13 years. The main cause of hilar obstruction was cholangiocarcinoma (32.9 %) followed by lymph node metastasis (23.2 %). Technical success was achieved in 75 patients (91.5 %), and clinical success in 45 patients (60 %). The authors found a significant correlation between clinical success rate when at least 50 % of viable parenchyma was drained (p = 0.016; OR = 4.15, 95 % CI 1.4–12.5). Considering liver sectors, higher clinical success rates were found when at least 2 sectors were drained (p < 0.001; OR = 8.50, 95 % CI 2.7–26.7). The correlation between unilateral versus bilateral drainage and clinical success was not statistically significant.

**Conclusion:**

Drainage of at least 50 % of volume hepatic parenchyma was associated with better outcomes as well as drainage of at least 2 hepatic sectors, regardless of if unilateral or bilateral.

## Highlights

• The main cause of hilar obstruction was cholangiocarcinoma (32.9 %) followed by lymph node metastasis (23.2 %).
• Technical success was achieved in 91.5 %, clinical success in 60 %.
• A significant correlation between clinical success rate was found when at least 50 % of viable parenchyma was drained (p = 0.016; OR = 4.15, 95 % CI 1.4–12.5).
• Considering liver sectors, higher clinical success rates were found when at least 2 sectors were drained (p < 0.001; OR = 8.50, 95 % CI 2.7–26.7).
• Correlation between unilateral versus bilateral drainage and clinical success was not statistically significant.

## Introduction

Malignant Hilar Biliary Obstruction (MHBO) usually presents in advanced-stage disease with a poor prognosis. Surgical treatment is possible in less than 20 % of patients and the 5-year survival rate is lower than 10 %.^1^ Effective biliary drainage is essential for the beginning of palliative chemotherapy. Palliative biliary drainage is usually obtained by percutaneous and/or endoscopic techniques. However, the best algorithm approach is a matter of debate.

One major debate regarding the endoscopic management of malignant hilar obstruction is whether bilateral is better than unilateral drainage. Several meta-analyses showed no difference in the clinical success rates comparing unilateral versus bilateral drainage,^2^, ^3^, ^4^ although a prospective randomized trial favored bilateral drainage.^5^ In some cases, the insistence on placing bilateral stents, prolonging anesthetic time, and increasing the risk of injection of contrast in areas that are not amenable to endoscopic drainage, can increase the risk of cholangitis.^6^

Regardless of the access technique, it is known that to achieve the desired clinical results with biliary drainage, effective drainage of significant liver parenchyma is necessary. The first study carried out in the late 80s suggested that drainage of 25 % of the liver parenchyma would be the minimum necessary for the relief of jaundice in patients without cholangitis.^7^ Recent studies have suggested that drainage of at least 50 % of the liver parenchyma is necessary to achieve a more significant reduction in bilirubin and longer survival, especially in patients with Bismuth III or IV malignant strictures.^8^ In addition, it is essential to consider factors such as atrophy of liver parenchyma, the volume of parenchyma involved by tumor, and liver dysfunction, which significantly impact the results of biliary drainage.^8^^,^^9^

Although the calculation of liver volumes using CT-scan is simple and may help in deciding which portion of the liver should be drained, this analysis is not routinely input in standard radiologic reports.

The primary outcome of this study was to correlate the clinical success rate of endoscopic drainage of malignant hilar strictures with the volume of drained liver calculated by CT scan. Secondary outcomes were to correlate clinical success rate with the number of hepatic sectors drained and unilateral or bilateral drainage.

## Methods

This was a retrospective study including all patients with malignant hilar obstruction who underwent Retrograde Endoscopic Cholangiography (ERCP) for biliary drainage at our unit, from January 2014 to December 2018, with Bismuth classification ^10^ type II, III or IV, and CT-scan performed within 2-weeks before the procedure. Patients with incomplete laboratory results, benign stenosis, Bismuth I and previous hepatectomy were excluded from the analysis. This study was approved by the ethical committee and review board of our institution NP038/14, in October 2018 and data was subsequently collected for analyses.

Laboratory exams and pre-ERCP clinical condition were properly recorded, including a history of previous biliary interventions, assessment of the clinical stage of the underlying neoplastic disease and performance status by the Eastern Cooperative Oncology Group (ECOG).^11^

### Assessment of liver volume

**Fig 1:**
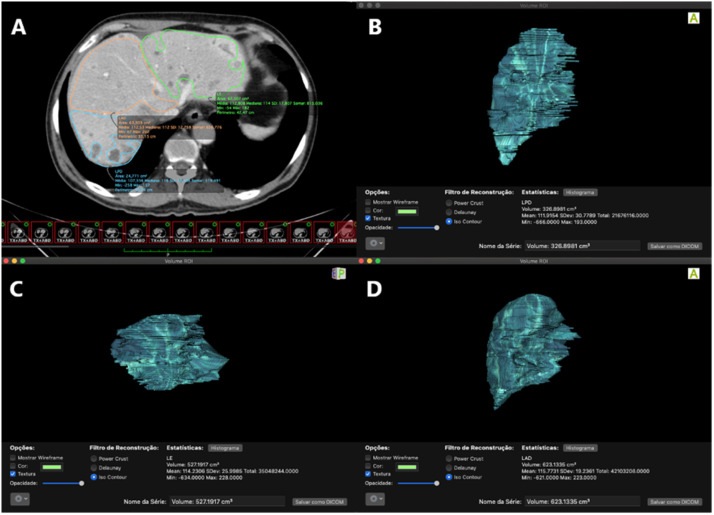
(A) Pre-drainage CT axial section, with selected areas for the left lobe and anterior and posterior segments of the right lobe (note that areas with metastatic lesions were excluded); (B) Volumetric calculation of the posterior segment of the right lobe; (C) Left lobe volumetric calculation; (D) Volumetric calculation of the anterior segment of the right lobe.

Assessment of liver volume was made by analysis of pre-drainage abdominal CT-scan. First, liver parenchyma was divided into 3 main sectors according to the distribution of hepatic vein branches: left lobe (segments II, III and IV), right anterior (segments V and VIII) and right posterior (segments VI and VII).^12^ Then, the area of each sector was calculated by delimitation of Regions of Interest (ROI) where each sector was delimited in multiple frames, thus making possible the closest volumetric calculation. For volumetric analysis, the software Osiris MD (Pixmeo SARL, Switzerland) was used. The tumoral volume was excluded from the volume of its respective liver segment. Thus, only functional liver areas were considered for the analysis (Fig. 1).

The Bismuth classification ^10^ was defined through a combined analysis of CT and/or Magnetic Resonance Cholangiography (MRC) when available, pre-ERCP, complemented by cholangiography findings during the endoscopic procedure.

### Procedures

All ERCP procedures were done under general anesthesia. Before ERCP, all patients with MHBO had their MRC and CT scans carefully analyzed, and the intention was always to achieve relief of biliary obstruction considering the amount of viable liver parenchyma and the segments mainly involved by biliary obstruction. The contrast was injected only when the guidewire was successfully advanced across the stricture. Dilation of hilar stricture before stent insertion was done at the discretion of the endoscopist performing the procedure. Antibiotic therapy was given routinely at the beginning of anesthesia.

### Assessment of liver sectors drained during ERCP

For the assessment of which sector(s) was(were) drained during ERCP, the authors analyzed the possible anatomical biliary variants.^13^^,^^14^ The most frequent variant is the right anterior hepatic duct joining the right posterior hepatic duct before the major confluence. In this case, a single stent inserted into the main right hepatic duct was considered as drainage of two sectors, in Bismuth II and IIIb tumors. In the case of one right hepatic branch (anterior or posterior) joining the left hepatic duct before major confluence, one stent inserted on the main left hepatic duct was considered sufficient to drain two sectors in Bismuth II. In variants where the right posterior duct is inserted in the confluence (absence of main right hepatic duct), one single stent would always drain only one sector.

### Assessment of liver volume drained

Assessment of liver volume drained was based on the relationship between the liver segment(s) that was/were drained during ERCP and the preoperative CT-scan volume of each segment.

### Definitions

Technical success was defined as the placement of plastic or metallic stents above the hilar stricture, draining at least the hepatic lobe with the major dilation on previous CT findings and the segments filled with contrast during ERCP. Clinical success was defined as a 50 % decrease or normalization of direct bilirubin level within 2 weeks of drainage, according to Tokyo Criteria 2014.^15^ Cholangitis was diagnosed according to Tokyo Guidelines 2018: evidence of systemic inflammation (fever, elevated C-reactive protein or leukocytosis), signs of cholestasis (jaundice and/or laboratorial abnormal liver function tests) and biliary dilation or evidence of the etiology on imaging studies were present.^16^

### Statistical analysis

The data were stored in an Excel® spreadsheet and imported into SPSS® 23.0 for MAC software. Categorical data were described by their absolute frequency and their respective proportion within the variable classifications. The distribution of continuous data was analyzed using the Shapiro-Wilk test. The Chi-Squared test or Fisher's exact test of contingency table were used for the analysis of categorical data. Mann-Whitney test was used for analysis of continuous data. The magnitude of the chance of success was described by the Odds Ratio when individuals with different “drainage levels” were compared. A statistically significant difference was considered when p < 0.05. Specificity and sensitivity were analyzed by the Receiver Operating Characteristic (ROC) curve, comparing clinical success and volume of drained liver.

### Results

A total of 82 patients met the inclusion criteria, 58.5 % female, with a mean age of 60 ± 13 years. The main cause of hilar obstruction was cholangiocarcinoma (33 %) followed by lymph node metastasis (23.2 %) and gallbladder carcinoma (14.6 %). The majority of patients had a good performance status (72 % ECOG ≤ 2) and the median Total Bilirubin (TB) before the procedure was 11.6 (1.15‒36.5 mg/dL). Thirty-two patients (39 %) had already been submitted to previous endoscopic drainage (34.1 % had plastic and 4.9 % had metal stents). Demographic data are shown in Table 1.

**Table 1:** Baseline characteristics of patients who underwent endoscopic biliary drainage with malignant hilar stricture.

	**Overall ( %)**
**Gender**	
Female	48 (58.5)
Male	34 (41.5
**Biliary type**	
Bismuth II	19 (23.2)
Bismuth III a	11 (13.4)
Bismuth III b	13 (15.8)
Bismuth IV	39 (47.6)
**Causes of biliary obstructions**	
Cholangiocarcinoma	27 (33)
Lymph node metastasis	19 (23.2)
Gallbladder carcinoma	12 (14.6)
Others	24 (29.2)
**ECOG**	
0	6 (7.3)
1	29 (35.3)
2	24 (29.2)
3	17 (20.7)
4	4 (4.9)
Not evaluated	2 (2.4)
**Indications**	
Cholangitis	24 (29.3)
Palliative drainage	47 (57.3)
Stent dysfunction	11 (14.4)
**Previous stent**	
None	50 (61)
Plastic	28 (34.1)
Metallic	4 (4.9)
**Serum Bilirubin before the procedure (mg/dL)**	
Total	11.6 (1.1‒36.5)
Direct	8.2 (0.58‒20)
Previous Chemotherapy	49 (59.7)

Technical success was achieved in 75 patients (91.5 %). Metallic stent was placed in 18 patients (24 %), plastic stent in 55 (73.3 %) and 2 patients (2.7 %) received both plastic and metallic stents. Drainage of at least 33 % of liver parenchyma was achieved in 64/75 patients (85.3 %). A drainage of at least 50 % of the liver parenchyma was achieved in 55 (73.3 %), and in 34 (45.3 %) patients it was possible to drain 100 % of the parenchyma.

Clinical success was observed in 45 patients (60 %). Most frequent adverse event was stent obstruction (16 %), followed by cholangitis (13.3 %). Descriptive data are available in Table 2.

**Table 2:** Descriptive characteristics of endoscopic biliary drainage in malignant hilar stricture.

	**Overall ( %)**
**Technical Success**	75 (91.5)
**Stent type**	
Metallic	18 (24)
Plastic	55 (73.3)
Metallic +Plastic	2 (2.7)
**Drained area**	
1 sector	23 (30.7)
> 1 sector	52 (69.3)
**Drained viable hepatic parenchyma ( %)**	
> 33 %	64 (85.4)
> 50 %	55 (73.3)
> 67 %	45 (60)
100 %	34 (45.3)
**Drained side**	
Unilateral	39 (52)
Bilateral	36 (48)
Clinical Success	45 (60)
**Adverse Events**	
Cholangitis	10 (13.3)
Pancreatitis	4 (5.3)
Cholecystitis	1 (1.3)
Stent Obstruction	12 (16)

### Correlation between drained hepatic volume and clinical success

The median hepatic volume drained of patients with clinical success was higher than patients with clinical failure, 100 % vs. 51.5 % respectively (p < 0.01 – Mann-Whitney test).

**Fig 2:**
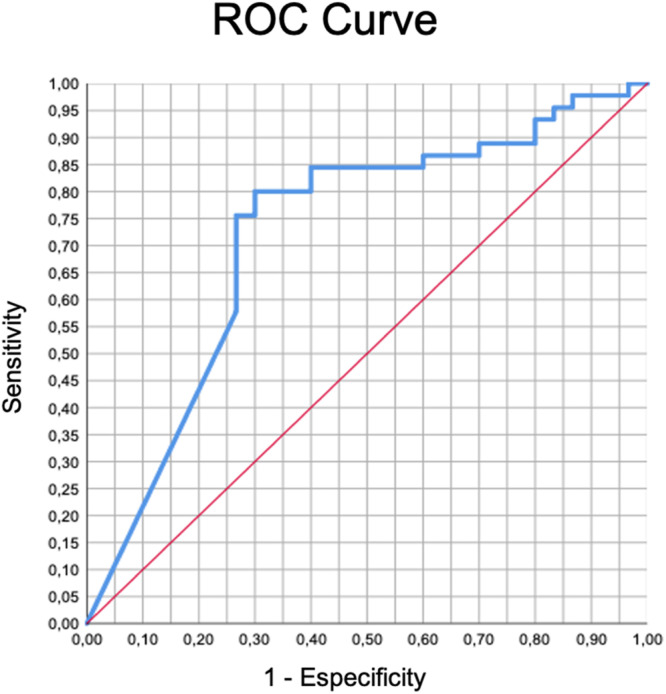
ROC curve analysis. Specificity and sensitivity were analyzed correlating clinical success with the volume of drained liver. Drainage of 67 % of liver parenchyma presented the higher area under the curve (72.07 [95 % CI 59.7 to 84.4], p < 0.01).

A significant correlation of clinical success with a percentage of drained hepatic volume was found when at least 50 % of viable parenchyma was drained (p = 0.016; OR = 4.15, 95 % CI 1.4–12.5). Using the ROC curve for sensitivity and specificity analysis, the authors observed that the drainage of 67 % of liver parenchyma presented the higher area under the curve correlating with clinical success (72.07 [95 % CI 59.7 to 84.4], p < 0.01 – Fig. 2).

### Correlation between the quantity of hepatic sectors drained unilateral or bilateral drainage and clinical success

Considering the division in hepatic sectors, it was possible to drain at least 2 sectors in 52 patients (69.3 %). A higher rate of clinical success was found when at least 2 sectors were drained (p < 0.001; OR = 8.50, 95 % CI 2.7–26.7).

Thirty-nine patients (52 %) received unilateral and 36 (48 %) received bilateral drainage. There was no correlation between unilateral versus bilateral drainage and clinical success (p = 0.17), (Table 3).

**Table 3:** Correlation of clinical success with a percentage of drained liver parenchyma, hepatic sectors and laterality.

**Variables**	**Total**	**Clinical success**		**OR [95 % CI]**^a^	**p**^b^
		**No**	**Yes**		
	**75 (100 %)**	**30 (40 %)**	**45 (60 %)**		
Percentage of drained parenchyma					
< 33 %	11 (14.7 %)	6 (54.5 %)	5 (45.4 %)	‒	-
≥ 33 %	64 (85.3 %)	24 (37.5 %)	40 (62.5 %)	2 [0.5‒7.5]	0.46
< 50 %	20 (26.7 %)	13 (65 %)	7 (35 %)		
≥ 50 %	55 (73.3 %)	17 (30.9 %)	38 (69.1 %)	4.15 [1.4‒12.5]	0.016
< 67 %	30 (40 %)	21 (70 %)	9 (30 %)	‒	‒
≥ 67 %	45 (60 %)	9 (20 %)	36 (80 %)	9.33 [3.1‒27.8]	<0.001
< 100 %	41 (54 %)	22 (53.7 %)	19 (46.3 %)	‒	‒
100 %	34 (45.3 %)	8 (23.5 %)	26 (76.5 %)	3.76 [1.3‒10.5]	0.016
Quantity of sectors drained					
1 sector	23 (30.7 %)	17 (73.9 %)	6 (26 %)	‒	‒
2 or more sectors	52 (69.3 %)	13 (25 %)	39 (75 %)	8.50 [2.7‒26.7]	<0.001
Laterality					
Unilateral	39 (52 %)	19 (48.7 %)	20 (51.3 %)	‒	‒
Bilateral	36 (48 %)	11 (30.5 %)	25 (69.4)	2.16 [0.82‒5.68]	0.17

a Considered significant p-values > 0.05.

b OR, Odds Ratio [95 % CI inferior to superior].

## Discussion

The endoscopic drainage of unresectable MHBO remains a major challenge with suboptimal clinical success results.^17^ The choice of stent, quantity, and location of drainage can make its placement even more challenging. For that reason, it is important to evaluate the aspects related to drainage responsible for clinical improvement, avoiding unnecessary over-manipulation.

The present study found similar rates compared to the current literature regarding technical success. Fang Yang et al., in a meta-analysis analyzing eight studies including 818 MHBO patients, found a technical success rate variation of 89.5 %–100 %.^3^ In this study, the technical success rate was 91.5 % without unilateral/bilateral and plastic/metallic stents distinction. When analyzing clinical success, the result was lower than expected (60 %). This might have occurred due to the high frequency (47 %) of type IV Bismuth strictures, which is a predictor of failure in endoscopic drainage.^17^

Although many authors suggested that drained hepatic volume might correlate with clinical success, only a few studies have directly approached this topic. Vienne et al. showed that the rate of effective drainage was significantly higher, with less cholangitis and longer survival (119 vs. 59 days) when more than 50 % of viable hepatic volume was drained in malignant hilar biliary obstruction and that inserting a stent in an atrophic sector was inefficient and increased the risk of cholangitis and should be avoided.^8^ The authors found a significant difference in clinical success when at least 50 % of hepatic volume was drained which corroborates Vienne et al. findings. More importantly, the drainage of 67 % of hepatic parenchyma presented the best correlation with clinical success, showing the importance of draining at least 2 liver sectors. Takahashi et al. found that drainage of at least 33 % of liver volume in patients with preserved liver function and 50 % or more in patients with impaired liver function significantly correlates with effective biliary drainage in MHBO.^9^ In this study, the stratification of groups according to liver function was not performed. This might be the reason for the clinical success in patients with drainage of at least 33 % of hepatic parenchyma was not statistically superior. Another interesting study demonstrated that draining 71 % or more of the liver segments was a significant predictor of chemotherapy and/or radiotherapy administration (92 % vs. 44 %, p = 0.0008) and correlated with survival rate. In addition, survival continued to improve as the liver drainage percentage increased beyond 50 %. Of note, the authors did not evaluate the volume of drained hepatic parenchyma.^18^

All these data support the assessment of volumetrics prior to ERCP. This allows us to better plan the procedure to ensure that at least 50 % of the functional parenchyma is drained, leading to improved outcomes, particularly in patients with liver impairment. The determination of the hepatic segments related to each liver sector by the analysis of pre-procedure CT-scan and cholangiography findings is possible and can facilitate and improve clinical results when CT volumetry is not available. It is reasonable to assume that the drainage of two or more liver sectors will drain at least 50 % of hepatic parenchyma. In the present study, drainage of at least 2 sectors accounted for significant improvement in clinical success rates (p < 0.001). The same result was not achieved when comparing unilateral and bilateral stenting, compatible with current literature that shows dissonant results in this subject.^2^, ^3^, ^4^, ^5^ This corroborates the thought that what matters for clinical success is the amount of drained functioning liver volume, regardless of unilateral or bilateral.

This study has some limitations. First, due to its retrospective nature, drainage approaches were not uniform. It depended on the endoscopist evaluation and patient status, as well as the availability of stents which might have led to performance bias. Secondly, this study was performed in a tertiary cancer hospital, with a biased sample of advanced cases, which could have contributed to the lower-than-expected clinical success outcomes. Other factors such as liver impairment and chemotherapy regimens were not in the scope of this study and were not assessed. Third, the evaluation of Bismuth types was partially made by cholangiography images, which can be difficult due to the low quantity of intended contrast injection. Finally, in this study, other drainage methods as percutaneous transhepatic or EUS-guided biliary drainage were not evaluated.

In conclusion, this study suggests that drainage of at least 50 % of hepatic parenchyma volume is associated with better outcomes as well as drainage of at least 2 hepatic sectors. There was no correlation between unilateral or bilateral drainage and clinical success.

## Authors’ contributions

Study concept and design: Bruno Costa Martins, Ernesto Q. Mendonça; Acquisition of data: Ernesto Q. Mendonça, Jennifer Nakamura Ruas; Analysis and interpretation of data: Bruno Costa Martins, Jennifer Nakamura Ruas, Fauze Maluf-Filho; Drafting manuscript: Bruno Costa Martins, Jennifer Nakamura Ruas, Fauze Maluf-Filho. All authors participated in the critical review and approval of the final draft submitted.

## Funding

There was no funding or financial support that could have influenced its outcome.

## Declaration of competing interest

The authors declare no conflicts of interest.
